# Supplementary material for: Content-rich biological network constructed by mining PubMed abstracts
Source: BMC Bioinformatics. 2004 Oct 8;5:147. doi: 10.1186/1471-2105-5-147 (PMC528731; doi:10.1186/1471-2105-5-147)
Supplement: Additional File 2 — The original results of the above study (non-essential files are deleted to keep the file size under the limit set by BMC bioinformatics). [file 1471-2105-5-147-S2.bz2 › chilibotAdditionalFile2/dip20/110ID8604139E454/html/FLIM_FLIN.html]

 


 **FLIM** and **FLIN** 
  
Found 43 abstracts in PubMed, retrieved 20.  
 

 What does Google say? 
 PDF only 
| .edu only 

---

**Interactive relationship** (e.g. stimulation, inhibition, etc)

**Neutral relationship**- Here, we have used affinity blotting to obtain direct evidence of interaction between Salmonella typhimurium  **FliM**  and  **FliN** ,  **FliM**  and FliG, and  **FliM**  and CheY P.  Ref: 9356251 J Mol Biol, 1997
- Distinct regions of bacterial flagellar switch protein  **FliM**  interact with FliG,  **FliN**  and CheY.  Ref: 9356251 J Mol Biol, 1997
- Two other proteins,  **FliM**  and  **FliN** , are known to bind to FliG and have also been suggested to be involved to some extent in torque generation.  Ref: 9573160 J Bacteriol, 1998
- Complementation analysis of this  **fliM**  mutant indicated that  **fliM**  and  **fliN**  transcription starts beyond the 5 end of fliK and terminates after  **fliN** .  Ref: 9683497 J Bacteriol, 1998
- Genetic analyses and binding studies have shown that  **FliM**  interacts with several other flagellar proteins, including  **FliN** , FliG, phosphorylated CheY, other copies of  **FliM** , and possibly MotA and FliF.  Ref: 9791106 J Bacteriol, 1998

**Non-interactive relationship** (e.g. studied together, co-existance, homology, etc.)

- In this work, the genes that encode the  **FliM**  and  **FliN**  proteins of Rhodobacter sphaeroides were characterized.  Ref: 9683497 J Bacteriol, 1998
- Electron microscopic analysis indicated that polar and lateral flagellum specific FliG,  **FliM** , and  **FliN**  switch proteins are necessary for assembly of the respective flagella.  Ref: 11948156 J Bacteriol, 2002
- The genes identified include flbD, motA, motB, fliL,  **fliM** ,  **fliN** , fliZ, fliP, fliQ, fliR, flhB, flhA, flhF and flbE.  Ref: 9168127 Gene, 1997
- The FliG,  **FliM** , and  **FliN**  proteins of the bacterial flagellar motor are believed to interact with one another to form the switch complex, which in turn is thought to interact with one of the chemotaxis proteins, CheY.  Ref: 9356251 J Mol Biol, 1997
- Four mutants had the KmR cassette inserted in genes possibly involved in flagella motor function pflA,  **fliM**  and orf1 downstream of the  **fliN**  gene.  Ref: 10463181 Microbiology, 1999
- The flagellar motor switch complex, consisting of the three proteins FliG,  **FliM** , and  **FliN** , plays a central role in bacterial motility and chemotaxis.  Ref: 10809678 J Bacteriol, 2000
- These basal bodies lacked C rings but had intact M rings, suggesting that  **FliM**  and  **FliN**  are part of the C ring but not a detectable part of the M ring.  Ref: 11591685 J Bacteriol, 2001
- Antisera to Escherichia coli and Salmonella typhimurium  **FliN** ,  **FliM** , FlhB and FlhA reacted with proteins of the predicted molecular weights in cell lysates of Bb.  Ref: 9168127 Gene, 1997
- None of the conserved acidic residues of MotA, FliG,  **FliM** , or  **FliN**  proved essential for torque generation.  Ref: 9573160 J Bacteriol, 1998
- Incubation of purified basal bodies at pH 4.5 removed  **FliM**  and  **FliN**  but not FliG or FliF.  Ref: 11591685 J Bacteriol, 2001
- Incubation of basal bodies at pH 2.5 removed FliG,  **FliM** , and  **FliN**  but not FliF.  Ref: 11591685 J Bacteriol, 2001
- To address this question, we purified the intact switch complex constituting the switch proteins FliG,  **FliM** ,  **FliN**  and the scaffolding protein FliF in quantities sufficient for biochemical work and used it to investigate whether or not the binding of CheY to the switch complex is cooperative.  Ref: 12736245 J Biol Chem< MedlineTA>J Biol Chem, 2001
- The Agrobacterium tumefaciens motor gene, motA, is in a linked cluster with the flagellar switch protein genes, fliG,  **fliM**  and  **fliN** .  Ref: 9161425 Gene, 1997
- The basal body C rings, composed of the  **FliM** , FliG and  **FliN**  proteins, form the rotor of the flagellar motor 2.  Ref: 10959841 Curr Biol< MedlineTA>Curr Biol, 1997
- FliG,  **FliM** , and  **FliN** , key proteins for torque generation, are located in two rings.  Ref: 10468575 Proc Natl Acad Sci U S A, 1999
- We have also examined the ability of various  **FliM**  deletion and truncation mutant proteins to bind to  **FliN** , FliG, and CheY P.  Ref: 9356251 J Mol Biol, 1997
- We propose a model in which the N terminal region of  **FliM**  binds to CheY P, the middle region of  **FliM**  binds to FliG, and the C terminal region binds to  **FliN** .  Ref: 9356251 J Mol Biol, 1997
- The flagellar switch genes  **fliM**  and  **fliN**  of Rhodobacter sphaeroides are contained in a large flagellar gene cluster.  Ref: 9683497 J Bacteriol, 1998
- Both  **FliM**  and  **FliN**  possess charged domains not found in proton type homologs.  Ref: 10648530 J Bacteriol, 2000
- In this work, we identify the switch components, FliG,  **FliM** , and  **FliN** , of the sodium type motor.  Ref: 10648530 J Bacteriol, 2000
- Here, we examine the effects of a set of linker insertions and partial deletions in  **FliM**  on its binding to  **FliN** , FliG, CheY, and phospho CheY and on its functions in flagellar assembly and rotation.  Ref: 9791106 J Bacteriol, 1998
- In order to elucidate the process of assembly of cytoplasmic substructures, the membrane localization of each component of the switch complex FliG,  **FliM** , and  **FliN**  in various nonflagellated mutants was examined by immunoblotting.  Ref: 9006037 J Bacteriol, 1997
- Phylogenetic analysis indicates that the polar and lateral FliG,  **FliM** , and  **FliN**  switch proteins are closely related and most likely arose as a gene duplication event.  Ref: 11948156 J Bacteriol, 2002
- The switch complex is composed of three proteins, FliG,  **FliM** , and  **FliN** .  Ref: 9727015 J Biol Chem, 1998
- FliG,  **FliM** , and  **FliN** .  Ref: 9774535 J Struct Biol, 1998
- Flagella purified from Salmonella enterica serovar Typhimurium contain FliG,  **FliM** , and  **FliN** , cytoplasmic proteins that are important in torque generation and switching, and FliF, a transmembrane structural protein.  Ref: 11591685 J Bacteriol, 2001
- FliG does not require  **FliM**  and  **FliN**  for this association, but  **FliM**  and  **FliN**  associate cooperatively with the membrane only through FliG.  Ref: 9006037 J Bacteriol, 1997
- We report the sequence of 3978 bp of the Agrobacterium tumefaciens chromosome which contains a putative operon encoding the homologues of the transmembrane proton channel protein MotA, and the flagellar switch proteins  **FliM** ,  **FliN**  and FliG.  Ref: 9161425 Gene, 1997
- About 25 copies of FliG are present in a large rotor mounted assembly termed the switch complex, which also contains the proteins  **FliM**  and  **FliN** .  Ref: 12093724 EMBO J, 2002
- Three Salmonella typhimurium flagellar motor proteins, FliG,  **FliM**  and  **FliN** , are required for the switching of rotation sense.  Ref: 10788321 J Mol Biol, 2000
